# Pathogenesis of Fungal Infections in Cystic Fibrosis

**DOI:** 10.1007/s12281-016-0268-z

**Published:** 2016-12-07

**Authors:** Craig Williams, Ranjith Ranjendran, Gordon Ramage

**Affiliations:** 1 Institute of Healthcare Policy and Practice, University of West of Scotland, Paisley, UK; 2 Oral Sciences Research Group, Glasgow Dental School, School of Medicine, Dentistry and Nursing, College of Medical, Veterinary and Life Sciences, University of Glasgow, Glasgow, UK

**Keywords:** Fungal infections, Cystic fibrosis, Pathogenesis, *Pseudomonas aeruginosa*, Review

## Abstract

For a long time, the microbiology of cystic fibrosis has been focussed on *Pseudomonas aeruginosa* and associated Gram-negative pathogens. An increasing body of evidence has been compiled demonstrating an important role for moulds and yeasts within this complex patient group. Whether or not fungi are active participants, spectators or transient passersby remain to be elucidated. However, functionally, they do appear to play a contributory role in pathogenesis, albeit we do not know if this is a direct or indirect effect. The following review examines some of the key evidence for the role of fungi in CF pathogenesis.

## Introduction

Cystic fibrosis (CF) is a systemic multi-organ, chronic condition, which causes functional disorders of the exocrine glands, which are located mainly in the respiratory and alimentary systems. It is the most common monogenetic autosomal recessive disease in Northern Europe and is caused by mutations of the CFTR gene located on the long arm of chromosome 7. Functionally, it codes for a chloride channel that, together with sodium channels, maintains the ion balance of epithelial cells within the exocrine glands, and is responsible for maintaining airway homeostasis and mucociliary clearance [70]. The CFTR mutation results in reduced secretion of chloride ions in cells leading to increased water absorption and viscid secretions, leading to defective mucociliary clearance, which ultimately drives the morbidity and mortality in the CF population due to the irreversible decline in lung function caused by microbial colonisation of the airways and the resulting overactive neutrophilic immunological response. This chronic bronchopulmonary disease leads to frequent hospitalisations and ultimately death.

In CF pathogenesis, the ‘vicious cycle’ of inflammation results from colonisation/infection of the respiratory tract, which progresses in an age-related manner. *Staphylococcus aureus* and *Haemophilus influenzae* begin to colonise in childhood or early adolescence, and as age increases, *Pseudomonas aeruginosa*, *Burkholderia cepacia complex* and non-tuberculous mycobacterial infection occur. Fungi are another group of microorganisms commonly found in clinical specimens from CF patients [49, 62]. However, the isolation of yeasts and moulds from CF patients is considered by some to be of secondary importance when compared to bacterial pathogens. In the recent years, *Aspergillus* spp., *Scedosporium* spp., *Exophiala* spp. and *Candida* spp. have all been isolated from different cohorts of CF patients [8, 13, 15, 35]. *A. fumigatus* has a prevalence rate of between 10 and 57% [4, 62], though other *Aspergillus* species have been isolated from the lungs including *A. niger*, *A. flavus*, *A. nidulans* and *A. terrus* [16, 80], as well as several yeasts such as *C. albicans*, *C. glabrata*, *C. krusei* and *C. parapsilosis* [12, 46]. Recent next-generation sequencing analysis suggests the mycobiome is more diverse than we really appreciate, although whether these are active participants, spectators or transient passersby remain to be elucidated [37, 82].

Given the ubiquitous nature of fungi in the environment, with thousands of conidia being inhaled every day [69], the detection of fungi in respiratory samples creates clinical uncertainty. A positive culture may indicate specimen contamination during sampling or laboratory processing, transient or chronic colonisation or an active infection. This ambiguity is reflected in epidemiological studies where prevalence rates are variable with the reported prevalence ranging from 6 to 57% with factors, such as culture frequency, laboratory methodology and expertise, duration of monitoring and the patient population all contributing to the differences [9, 62]. Regardless of the cause, the number of patients with mould in sputum samples is increasing, exemplified in a cohort database study between 1997 and 2007 that reported an increase in the prevalence of filamentous fungi, predominantly *A. fumigatus* from 2 to 29% [80].

### The Clinical Problem

The lung mycobiome has been suggested to have a significant impact on clinical outcome of CF and other chronic respiratory diseases, such as asthma, chronic obstructive pulmonary disease and bronchiectasis [54]. The microbiology of lower respiratory tract is primarily associated with biofilm infection, with *P. aeruginosa* being a primary causative agent in CF [78]. It is becoming increasingly recognised however that fungal biofilms can persist in the lung and contribute to pathology [28, 67, 83]. Importantly, these structures are highly resistant to antifungal therapy, which complicates chemotherapeutic interventions [51, 74]. In the CF lung filamentous moulds such as *A. fumigatus* may cause a spectrum of respiratory disease, including allergic bronchopulmonary aspergillosis (ABPA), an aspergilloma and invasive aspergillosis (IA) [20].

A number of recent studies have reported that lung function declines more rapidly in patients chronically colonised with *A. fumigatus* [2, 44, 71] or co-infected with *A. fumigatus* and *P. aeruginosa* when compared to single species infection [56], a phenomenon also reported with *Candida* species and *P. aeruginosa* [13]. In addition, *A. fumigatus* is the only species that has been associated with an increased risk for the development of infection with *P. aeruginosa* [29]. Perhaps infection with fungi should be treated more seriously in terms of managing CF patients with combinations of antifungal and antibacterial drugs.

### Aspergillus Infection

Among the fungal species isolated from the airways of CF patients, the most frequent is *A. fumigatus*, but *A. flavus*, *A. niger*, *A. terreus* and *A. nidulans* are all reported and may cause sinusitis, bronchitis or allergic bronchopulmonary aspergillosis (ABPA) [12, 40]. *A. fumigatus* is a saprophytic spore-forming mould found widely in the environment [32]. The spores are 2–4 mm in diameter, and thousands of these spores are inhaled daily [38]. These inhaled spores settle the mucous membrane of the upper airways and the lungs [12], and either due to the thick mucus in the airways providing a source of nutrients supporting growth of the fungus or failure of mucociliary clearance the spores germinate. During fungal growth, the cells may secrete proteolytic enzymes (proteases, phosphatases), which inhibit their phagocytosis and further facilitate adhesion and colonisation within the airways [12, 68]. Another predisposing factor is the use of broad-spectrum antimicrobials in the management of patients with CF. Tobramycin used to treat *P. aeruginosa* infection encourages the colonisation of filamentous fungi *A. fumigatus* [12]. Though it has been suggested that antimicrobial management of this patient group with antibiotics is able to positively impact the reduction in bioburden of *Aspergillus* species, suggesting a complex inter-kingdom relationship is worth further exploration [7].

### Allergic Bronchopulmonary Aspergillosis

Allergic bronchopulmonary aspergillosis (ABPA) is the best recognised fungal disease in CF. It has a reported prevalence of 2–25% [3, 44] and is a result of a Th2-mediated hypersensitivity response to *Aspergillus* antigen. The current first-line treatment of ABPA is oral prednisolone [27]. While itraconazole is widely used as an adjunct to steroid therapy, azole resistance [10] is now a potential problem and there are no large studies available showing a direct benefit of using antifungals in the management of ABPA in patients with CF or any steroid-sparing effect. A case report and a small study of nebulised amphotericin B (AMB) in this condition suggests that nebulised liposomal AMB could represent a possible strategy in ABPA management in CF patients [11, 63], and in asthmatics with ABPA, a further small study suggests that nebulised amphotericin may be beneficial in decreasing the frequency of exacerbations in patients with ABPA complicating asthma [65]. Again, in asthmatics, ABPA has been shown to progress to invasive pulmonary aspergillosis which is a fulminant disease, and in a recent systematic review, only three of the nine patients survived [44]. This has not yet been described in patients with CF.

### Aspergillus Bronchitis

This was first described by Shoseyov et al. [75] who described a group of patients with CF who were experiencing respiratory exacerbations which were non-responsive to appropriate antimicrobial therapy, cultured *Aspergillus* spp. from sputum and responded to antifungal therapy [75]. Aspergillus bronchitis may be responsible for persistent respiratory symptoms in patients with CF in whom *Aspergillus* is detected repeatedly in sputum, but have no evidence of parenchymal disease and no hypersensitivity to *Aspergillus* [36]. While it is largely a diagnosis of exclusion, Chrdle et al. [14] have further characterised this patient population and reviewed 400 patients with a positive culture or real-time PCR for *Aspergillus* spp. Seventeen patients fulfilled the criteria for aspergillus bronchitis. Thirteen patients cultured *A. fumigatus*, 3 *A. niger*, and 1 *A. terreus*. Twelve patients had elevated aspergillus IgG, with a mean of 89.2 mg/L and five (29%) had elevated aspergillus precipitins. Fifty percent had a major response to antifungal therapy and five of 12 (42%) patients relapsed, requiring long-term therapy [14]. Elevated IgG without elevation of IgE in the presence of either positive aspergillus culture, PCR or galactomannan has been suggested as a diagnostic criterion for aspergillus bronchitis [6, 32].

### Aspergillus Biofilm

*Aspergillus* has been shown to produce biofilms both in vitro [50] and in vivo [41]. These are multicellular populations of filamentous intertwined hyphae attached to surfaces or one another and enclosed within a dense extracellular matrix (ECM) [67]. Microbes in biofilms have altered metabolism compared to the same organisms growing planktonically, and the ECM provides microbes with protection from host defences as well as tolerance to some antimicrobial drugs [51]. *Aspergillus* is recognised bronchoscopically by the production of mucus plugs, which may be due to the production of glycan polymers. The ECM includes the polysaccharides, galactomannan, galactosaminogalactan, α-1,3 glucans, monosaccharides, proteins, melanin and extracellular DNA [66, 84]. The *Aspergillus* galactosaminogalactan allows adherence to host constituents and conceals hyphal beta-glucan from the immune system [25, 26], and the extracellular DNA is important for protection from environmental stresses, including antifungal therapy [64]. These types of structures are critically important, particularly as they can support the growth and proliferation of bacterial species, while providing physical protections from environmental stressors [61].

### Inter-kingdom Interactions

The most commonly isolated microbial pathogen is *P. aeruginosa*, which has been reported to colonise the airways of around 50% of adult patients with CF [72]. This is the most prevalent and persistent microbe found in the CF lung [19], and is associated with a more rapid decline in lung function, increased hospitalisation and a decreased life expectancy [21, 55]. Infection in CF patients is also commonly associated *S. aureus and H. influenzae*, and recent advances in culture-independent, NGS technologies, have revealed that the microbiome of the CF lung is much richer than previously appreciated comprising of a diverse range of bacterial and fungal pathogens [82], of which *A. fumigatus* is the most prevalent filamentous fungi [67]. The lungs of CF sufferers are lined with a thick viscous mucus layer susceptible to polymicrobial infections, leading to recurrent infections and continuous inflammation [70]. The interplay between the pathogens residing in the lung may be responsible for the acute exacerbations associated with CF, where the balance is tipped towards an environment with excess inflammatory, oxidative and proteolytic activity. Several studies have identified an association between *A. fumigatus* and *P. aeruginosa*, whereby co-infection saw decreased pulmonary function in comparison to those with a mono-infection [2], a phenomenon also reported with *Candida* species and *P. aeruginosa* [13]. Evidence is therefore increasing for the need for improved clinical management of these patients [19]. Indeed, inter-kingdom interactions of the CF lung, and elsewhere, may lead to adverse clinical outcomes [39]. The ability of these microbes to form strong mixed species biofilms likely contributes towards their persistence, making it extremely difficult to eradicate the infection [42, 74].

The CF lung is a site of intense inter-kingdom interaction, where *P. aeruginosa* is a primary participant. It has been shown that *P. aeruginosa* is able to selectively form biofilms on *C. albicans*, hyphae, but not the yeast form which results in the death of the *Candida* [30]. Presumably, this occurs through the release of a phenazine toxin [23, 47]. It has also been shown to inhibit the morphological transition through a 3-oxo-C12 homoserine lactone [31], a phenomenon replicated in studies of *A. fumigatus* biofilm [52]. Recent evidence from a murine model demonstrated that lung tissue injury caused by *P. aeruginosa* infection is alleviated if preceded by a short-term *C. albicans* colonisation [1]. This was a result of *C. albicans* activating IL-22 producing innate lymphoid cells, which provided protection from *P. aeruginosa* induced injury [45]. Given the dynamic relationship between these organisms, it is not surprising that the release by *C. albicans* of its quorum sensing molecule farnesol impacts *P. aeurginosa* by inhibiting its quinolone signalling, which controls pyocyanin production [17]. These studies highlight the on-going and dynamic battle within a polymicrobial environment such as the CF lung, which clearly plays a crucial role in the overall pathogenesis of disease [61]. Elegant studies in a Drosophila fruit fly infection model of polymicrobial infection demonstrate this point. This showed that microorganisms of the CF airways were able to influence the outcome of an infection depending on the presence or absence of *P. aeruginosa* [76, 77].

*P. aeruginosa* has also been shown to inhibit *A. fumigatus* filamentation via the release of molecules involved in intra-cellular communication [52]. Investigations into the interactions between these two are limited; however, the release of small molecules designed to inhibit fungal growth appear to be the primary form of interaction. One particular group of metabolites known as phenazines have been reported to inhibit *A. fumigatus* biofilm formation; however, it was also found that *A. fumigatus* was able to convert these metabolites released by *P. aeruginosa* to produce fungal siderophores, which may in turn influence CF progression [48]. Furthermore, *P. aeruginosa* releases the metalloprotease elastase, which has been shown to be toxic to host cells [79]. It was found that elastase production was constitutive, but became significantly increased in the presence of *A. fumigatus* during biofilm co-culture. Furthermore, elastase was cytotoxic to human lung adenocarcinoma cells, and therefore, the presence of both of these pathogens could contribute towards enhanced pathogenicity [79]. Interestingly though, the inhibition of *A. fumigatus* is related to the source and phenotype of the *P. aeruginosa* isolate with CF isolates more inhibitory than non-CF isolates, and non-mucoid CF isolates most inhibitory suggesting a role for the extensive evolutionary changes in *P. aeruginosa* which have been described in association with chronic residence in CF airways and may reflect adaptive changes to life in a polymicrobial environment [22]. *P. aeruginosa* has also been known to suppress the growth of a number of other CF-related fungi such as *C. albicans*, and *Cryptococcus neoformans* and more recently *Scedosporium aurantiacum*, where inhibition of growth was observed only in co-cultures of *P. aeruginosa* and *S. aurantiacum* with the cell fractions failing to act against the fungus. In parallel with the observations on *Aspergillus*/*P. aeruginosa* interactions, the ability of *P. aeruginosa* to form biofilms was an important, although not crucial, factor in inhibiting the growth of *S. aurantiacum* in a lung-mimicking environment [33].

Thus, in general, evidence suggests that co-isolation of bacteria and fungi indicate a poorer prognosis. However, the relationship between the two kingdoms remains poorly understood and requires further investigation.

### Other Moulds

Members of the *Scedosporium* species complex are chronic colonisers and emerging pathogens in patients with CF [15]. Rates of isolation range from 3.1% in Germany where a selective agar was used [73] to 10.6% in Austria where selective agar and homogenisation of samples was employed [43]. While *Scedosporium apiospermum* has been reported in a single case as a cause of acute respiratory distress in a child with CF [58], neither colonisation with nor sensitization to *Scedosporium apiospermum* complex is associated with poorer lung function [60, 73]. However, allergic responses and risk of dissemination in immunocompromised hosts have been described. Interestingly, in comparison with *A. fumigatus*, patients colonised with this fungus are less likely to be co-colonised with *P. aeruginosa* [8]. There is geographical variation in isolation rates, but also a large discrepancy between relatively high isolation frequency (6.5–10%) in patients and low environmental abundance which raises questions about how initial acquisition actually occurs in patients with CF [85]. Genotype analysis of sequential isolates demonstrates that individual patients are colonised by unique phenotypes which are conserved over time [18].

### Yeasts

The rate of recovery of *Exophiala dermatitidis* from the airways of CF patients again varies from 4% in Germany to 17% in Sweden [34, 86]. In the Swedish study, patients with higher levels of *E. dermatitidis* IgG antibodies were more often colonised with non-tuberculous mycobacteria and had lower than predicted FEV1 %. Colonisation and infections with *E. dermatitidis* occur considerably more frequently in the group of patients with pancreas failure and with advanced disease [35]. Other mould species such as *Paecilomyces* spp., *Penicillium* spp., *Alternaria* spp. or *Cladosporium* spp. are seldom cultured from clinical material and probably have no impact on clinical symptoms or underlying disease.

*C. albicans* can be isolated from respiratory cultures in up to 75% of patients with CF [81], and the frequent use of antibiotics and inhaled steroids may predispose patients to colonisation with *Candida* species [53, 57]. There is still debate around the significance of the presence of *Candida* in the respiratory tract in patients with CF, though limited studies have suggested that chronic respiratory colonisation with *C. albicans* is associated with worsening of FEV1 in CF [13, 24]. This raises the possibilities that species derived from the oral cavity and usually considered as clinically insignificant may be pathogenic possibly due to a complex interaction between typical pathogens and microbiota [59]. However, no pathogenic mechanism has been postulated, and given the frequency of isolation of *Candida* species from respiratory samples, the association remains controversial. *Candida* also differs from *Aspergillus* in that *Candida* sensitization is not associated with greater lung function decline or pulmonary exacerbations [5], so overall further prospective studies are needed to confirm this association.

## Conclusions

Although our knowledge regarding the role of fungi in the pathogenesis of CF is improving, many questions remain. Are certain fungi pathogenic, and if so, is the mechanism of pathogenicity direct or mediated by complex interactions within the lung microbiome? If pathogenicity is accepted, should attempts be made to eradicate fungi along with antibacterial treatments? If so, what drugs should be used and for what duration? These questions are difficult to answer on the basis of existing knowledge, and further studies are needed both in individual fungi and in the context of a complex inter-kingdom microbiome.
